# Information diffusion in signed networks

**DOI:** 10.1371/journal.pone.0224177

**Published:** 2019-10-29

**Authors:** Xiaochen He, Haifeng Du, Marcus W. Feldman, Guangyu Li

**Affiliations:** 1 Center for Administration and Complexity Science, Xi’an Jiaotong University, Xi’an, China; 2 Department of Sociology, Cornell University, Ithaca, New York, United States of America; 3 Department of Biology, Stanford University, Stanford, California, United States of America; Universidad Rey Juan Carlos, SPAIN

## Abstract

Information diffusion has been widely discussed in various disciplines including sociology, economics, physics or computer science. In this paper, we generalize the linear threshold model in signed networks consisting of both positive and negative links. We analyze the dynamics of the spread of information based on balance theory, and find that a signed network can generate path dependence while structural balance can help remove the path dependence when seeded with balanced initialized active nodes. Simulation shows that the diffusion of information based on positive links contradicts that based on negative links. More positive links in signed networks are more likely to activate nodes and remove path dependence, but they can reduce predictability that is based on active states. We also find that a balanced structure can facilitate both the magnitude and speed of information diffusion, remove the path dependence, and cause polarization.

## Introduction

The analysis of information diffusion plays an important role in understanding and predicting information flows, and has been widely discussed in various disciplines including sociology, economics, physics and computer science [1–5]. One of the applications is the analysis of collective action, in which a small group of individuals have been initially deprived of a right and ask for the return of this right. Their behaviors may then influence their friends or colleagues, triggering a cascade in the social network.

A series of models have been proposed to analyze information diffusion in social networks, most of which are based on two fundamental models: the independent cascade (IC) model, in which diffusion is treated as a cascade of infection over the network [6], and the linear threshold (LT) model, in which the diffusion is based on thresholds of influence due to the neighborhood [7]. Along with IC and LT models, many other diffusion models have been constructed to satisfy different requirements. Kempe et al. [8] generalized Granovetter’s LT model and studied the influence maximization problem in IC and LT models. Chen et al. [9] generalized the IC model in considering the diffusion of negative opinions. Borodin et al. [10] proposed an extended LT model in which two opposing opinions compete to maximize the rate of diffusion. Lee et al. [11] proposed a continuously activated and time-restricted IC model where a node can activate its neighbor repeatedly. Wang et al. [12] proposed a weighted cascade model, which treats node attributes that are independent of the network structure. Based on the IC and LT, epidemic models such as SIR (susceptible-infected-removed) or SIS (susceptible-infected-susceptible) models and their generalizations have also been proposed [13–16]. A number of studies have focused on the diffusion over dynamic networks [17]. Snijders et al. [18] proposed a stochastic model where network edge and node attributes evolve simultaneously over time, based on which they explained the diffusion of alcohol consumption among adolescent friends. A subsequent study by Steglich et al. [19] built a statistical model to separate the effects of node changes from edge changes. Aral et al. [20] developed a method to distinguish the two kinds of effects in dynamic networks and applied this method to a real messaging network. Greenan then proposed an extension of this model to explore the diffusion of innovations with an example concerning the initiation of cannabis smoking among adolescents [21]. Apolloni et al. [22] discussed the spread of information via conversations in a dynamic simulated socio-technical network where the list of demographic characteristics and the amount of contact were both included to model change in nodes and edges. By allowing nodes and edges to join and leave the network, Gayraud et al. [23] extended the IC and LT to account for network evolution, and found that delaying the initiators’ activation can help increase diffusion. Compared with the random walk strategy, Guimarães et al. [24] found that using centrality can accelerate the diffusion process over dynamic networks. In addition, several diffusion models related to evolutionary game theory in dynamic networks have been also studied [25–27].

Most of the above models are constructed on non-negative networks, where the effect of non-negative relations is stressed, while the impact of negative relations has been ignored. In reality, information diffusion among individuals is not only spread by trusting or cooperating relationships, but may also be subject to relations that involve controversy or conflict [28]. For example, if you heard from some of your friends that they joined a club, then you might join too. But if someone you dislike or some of your opponents joined this club, then you might be less likely to join. Instead, you might join another club or not join any club. Several studies have pointed out that negative interactions also play important roles in collective dynamics [29–32].

Networks consisting of positive and negative relations are called signed networks, where the sign “+” denotes a positive relation and “–” denotes a negative relation [33]. These signed relations can simply signal positive or negative impact on information diffusion, and thus provide information that individuals may use to activate their states during the evolution of collective behaviors.

The theory of structural balance plays an important role in the evolution of signed networks [34]. IC or LT models consider how individual behaviors evolve to a collective behavior, while structural balance focuses on how an individual’s behavior can affect others’ behaviors as a result of social interaction. Combining structural balance with diffusion models may produce a more natural set of choices. In this paper, we focus mainly on diffusion over static networks, generalize the diffusion model in signed networks, and examine the impact of structural balance on the propagation. The rest of this paper is organized as follows. A linear threshold model for signed networks is proposed in section 2; section 3 explains the relationship between the threshold model and structural balance; simulations and experiments are shown in section 4, and our conclusions are presented in section 5.

## Linear threshold model in signed networks

According to Kempe’s LT, each individual can be in one of two states: active *s*_*v*_ = 1 or inactive *s*_*v*_ = 0. An individual *v* will be influenced by each of its neighbors *w* based on a weight *b*_*v*,*w*_ such that $\sum_{w}^{Neighbors\;of\;v}b_{v,w}=1$. Then $\sum_{w}^{Neighbors\;of\;v}b_{v,w}\cdot s_{w}$ can be regarded as the proportion of active neighbors. If the network does not have weights (*b*_*v*,*w*_ is 1/ number of *v*’s neighbors), then $\sum_{w}^{Neighbors\;of\;v}b_{v,w}\cdot s_{w}$ becomes the number of active neighbors of *v* divided by its total number of neighbors. Each node *v* will be assigned a threshold 0 ≤ *T*_*v*_ ≤ 1 uniformly at random, which represents the fraction of *v*’s neighbors that must become active in order to activate *v*. The dynamics of the process then proceed as follows. At each time *t*, every node *v* is selected once.

1. If *v*’s state is active, it will remain active;
2. If *v*’s state is inactive, but $\sum_{w}^{Neighbors\;of\;v}b_{v,w}\cdot s_{w}\geq T_{v}$, then it will become active.

**Fig 1:**
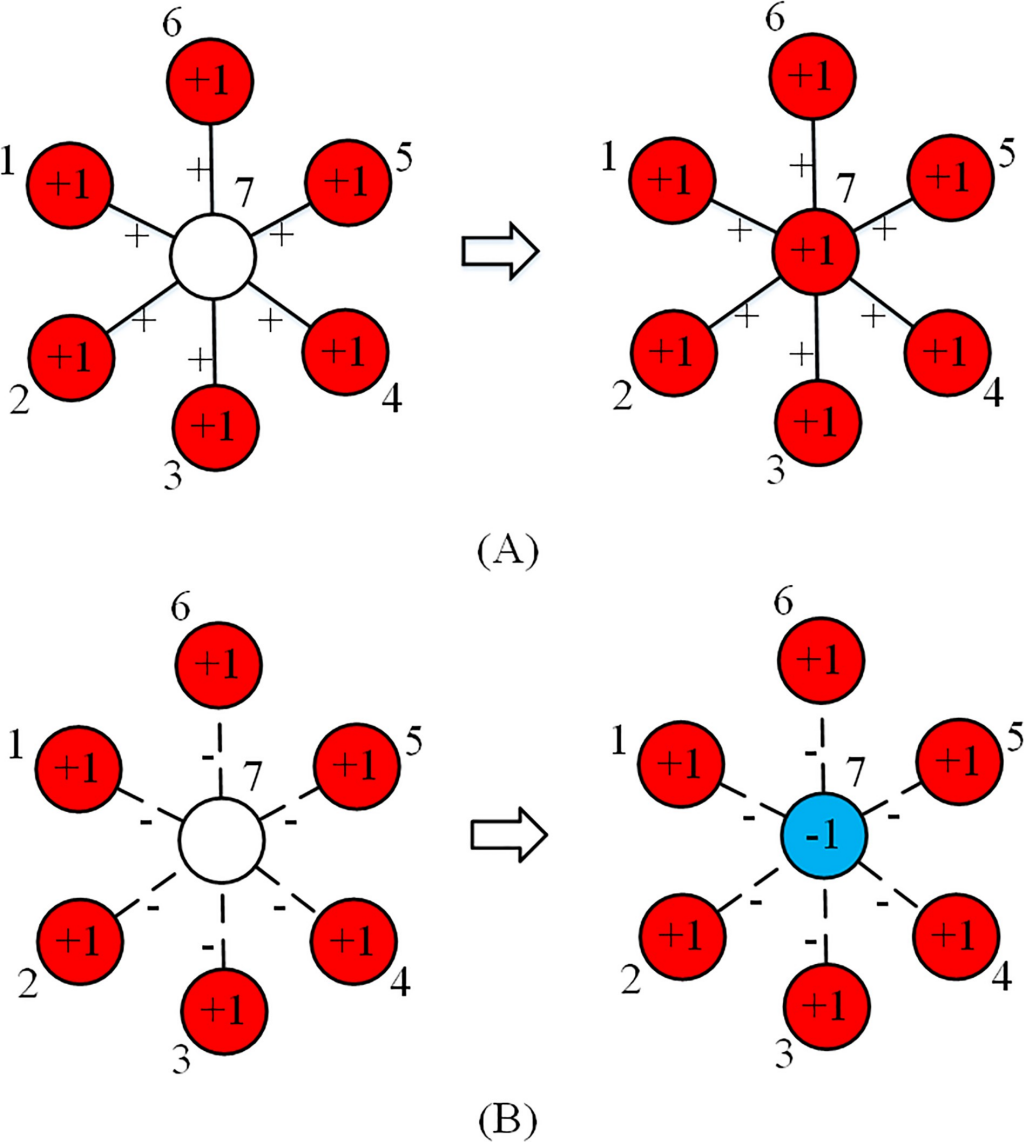
Activating nodes according to homophily and xenophobia. The red node represents an active +1 node, the blue node represents an active –1 node, while the white one represents an inactive node. With homophily, an inactive node can be activated to the same attribute as its friends as shown in (A), while it can be activated to the opposite attribute from its enemies according to xenophobia as shown in (B).

Compared with the classic LT model, negative relations in signed networks may generate a completely different mechanism, because a negative relation can not only impede the regular diffusion, but also result in diffusion of opposing information. A common phenomenon is that people tend to do the same things as friends, while keeping a distance from people who belong to other groups [31]. Actually, homophily and xenophobia play an important role in neighbors’ attributes in signed networks [35–38]. Fig 1 gives an example of how an individual is affected by its neighbors according to homophily and xenophobia. In Fig 1(A), if one node is located with all its friends, then it will follow these friends and behave like them, e.g., voting for a political leader. But if the node is located with all its enemies, as in Fig 1(B), then it will not follow its neighbors, and may vote for the opposing political leader instead. This is also consistent with social identity theory, according to which group identification may predict the likelihood of individuals’ behaviors in social change decisions [39–41].

In order to extend Kempe’s model to signed networks, we propose a new form of LT. In the real world, homophily and xenophobia can be observed in many types of interactions, including friendships, politics, international relations, etc. [42]. Since two nodes with a reciprocated tie are more likely to form a positive diffusion line, while those in conflict tend to construct a negative diffusion line, we design the linear threshold model as follows:

Each individual *v* can be in one of three states: *s*_*v*_ = 0, which represents the inactive state, *s*_*v*_ = +1, which represents the active state of an action, *s*_*v*_ = −1, which represents the active state of the opposite action. Individual *v* will be affected by each of its positive-link neighbors *w* based on a weight *b*_*v*,*w*_, while it may be influenced by each of its negative-link neighbors *u* based on a weight *λ* · *b*_*v*,*u*_, where *λ* represents the weight of the impact of negative relative to positive edges. The weight *b*_*v*,*w*_ will satisfy $\sum_{w}^{N_{pv}}b_{v,w}+\sum_{u}^{N_{nv}}\lambda \cdot b_{v,u}=1$ (*N*_*pv*_ is the number of *v*’s positive-link neighbors, while *N*_*nv*_ is the number of *v*’s negative-link neighbors). Each node *v* will be assigned a threshold 0 ≤ *T*_*v*_ ≤ 1, which represents the fraction of *v*’s neighbors that must become active in order to activate *v*. The dynamics of the process proceed as follows.

1. If *v*’s state is active, it will remain active in the same state, i.e., if it is +1 active, it will remain +1 active and if it is –1 active, it will remain –1 active;
2. If *v*’s state is inactive, it will activate to +1 if $\sum_{w}^{N_{pv}}b_{v,w}\cdot s_{w}-\sum_{u}^{N_{nv}}\lambda \cdot b_{v,u}\cdot s_{u}\geq T_{v}$ and will activate to –1 if $-(\sum_{w}^{N_{pv}}b_{v,w}\cdot s_{w}-\sum_{u}^{N_{nv}}\lambda \cdot b_{v,u}\cdot s_{u})\geq T_{v}$.

In this paper, we investigate normal diffusion on signed networks; the impacts of threshold distribution and link weight distribution on the dynamics will be studied in future work. Here we ignore these two effects and assign to each node the same threshold *T*, which determines the fraction of neighbors required to activate it, and we set the same link influence weight *b* for each node *v* from all its neighbors *i*, which can be computed as *b*_*vi*_ = 1/(*N*_*pv*_ + *λ* · *N*_*nv*_).

A single active node cannot initiate an information diffusion, i.e., a single active node cannot activate a second or third node because of the threshold, and thus the information cannot diffuse from the first step. So we randomly select a focal node to be activated with all its positive-link neighbors being activated to the same state, and all its negative-link neighbors being activated to the opposite state. Since activating a node to +1 and –1 have the same meaning in this paper, we activate the focal node to +1 in order to fix the initial state. The specific procedure for updating node status is shown in Algorithm1.

**Algorithm 1**. The algorithm for updating node status.

1. Input: The signed network matrix *A*. The effect of negative links *λ*. The threshold *T*. The maximum iterations *I*_*max*_.

2. The initial state of every node is inactive (*s* = 0), then randomly select one node and activate it to +1, while activating all of its positive-link neighbors to +1 and all its negative-link neighbors to –1 at the same time.

3. For *i* = 1; *i* ≤ *I*_max_; *i* + +

4. Randomly choose an inactive node *v*;

5. Compute the link influence weight for node *v b*_*v*_ = 1/(*N*_*pv*_ + *λ* · *N*_*nv*_) where *N*_*pv*_ is the number of *v*’s positive-link neighbors and *N*_*nv*_ is the number of *v*’s negative-link neighbors;

6. Compute *v*’s active energy $E_{v}=\sum_{w}^{N_{pv}}b_{v}\cdot s_{w}-\sum_{u}^{N_{nv}}\lambda \cdot b_{v}\cdot s_{u}$ based on its neighbors’ activated states;

7. If *E*_*v*_ ≥ *T*

8.  Activate *v* to +1, i.e. *s*_*v*_ = +1;

9. Else if *E*_*v*_ ≤ −*T*

10.  Activate *v* to –1, i.e. *s*_*v*_ = –1;

11. Else

12.  *v* remains inactive, i.e. *s*_*v*_ = 0;

13. End if

14.End for

15.Output: the activated state for each node.

**Fig 2:**
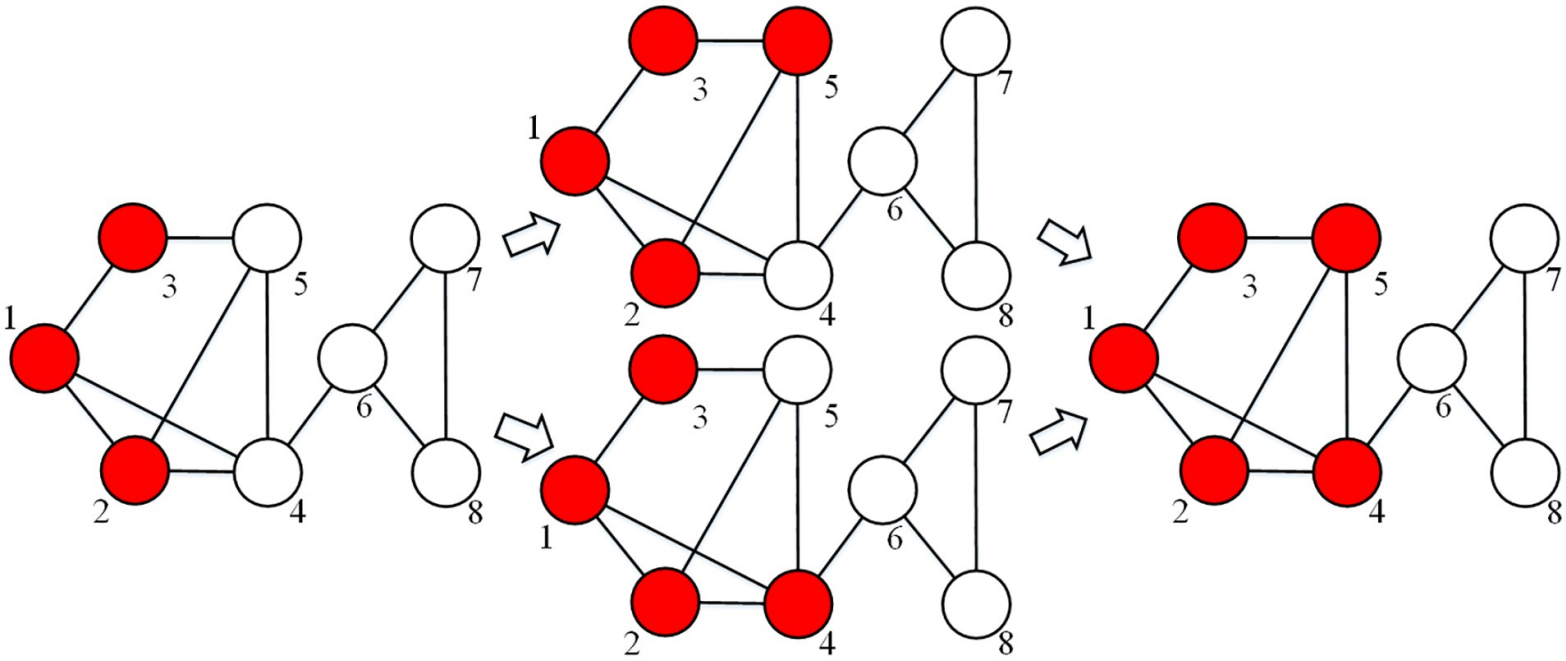
Diffusion processes of the classic LT. The red nodes are active and the white ones inactive. This figure shows that the diffusion in non-negative networks is predictable.

For the classic LT, once the contagion spreads from the initial active nodes through the network, the system eventually reaches a stationary configuration if the threshold, the network structure, the diffusion parameters, and the focal nodes are fixed. In other words, the classic LT is path-independent if nodes can be repeatedly chosen, e.g., a node that is not activated at this time may be activated at one time in the future. In this model, a path-dependent process entails that the diffusion result may vary for different paths of diffusion, while a path-independent process is one for which the diffusion result is always consistent no matter the path of diffusion. Fig 2 gives an illustration of path independence in the classic LT: if the threshold *T* is assigned to be 0.5, then it does not matter whether we first activate node 4 or node 5; the final result is the same with active nodes 4 and 5, and inactive nodes 6, 7, 8. However, it should be noted that LT may be path-dependent if inactive nodes can be only chosen once or a limited number of times.

**Fig 3:**
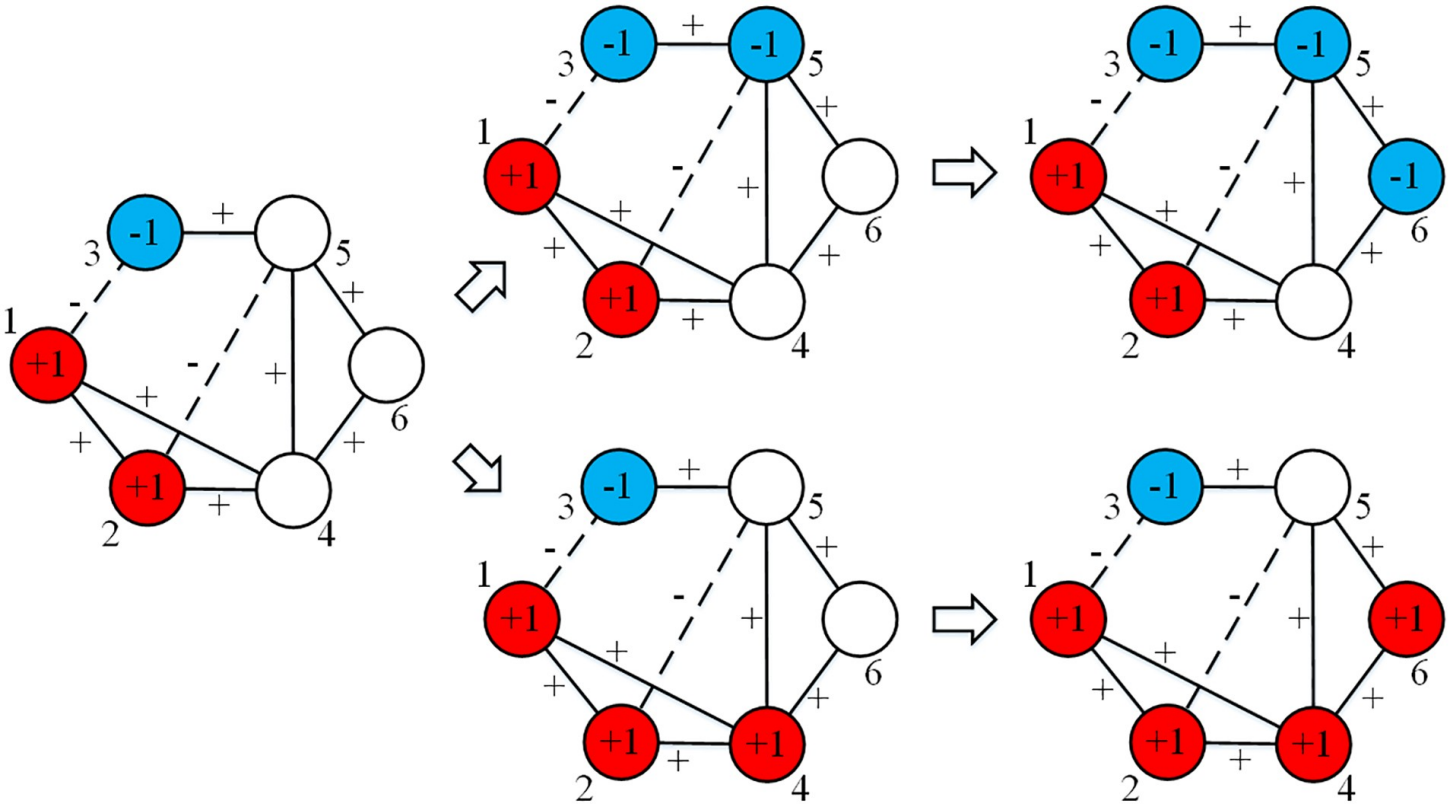
Diffusion processes of LT in signed networks. Red nodes represent active +1 nodes, blue nodes represent active –1 nodes, while white ones represent inactive nodes. This figure shows that the diffusion result in signed networks is unpredictable.

However, when we generalize LT to signed networks, things can be different. Even if we fix all the necessary parameters, the diffusion results cannot be predicted because diffusion by negative links will contradict that by positive links, which increases uncertainty in the dynamic process. As shown in Fig 3, if the threshold *T* is assigned as 0.5, and node 5 is first activated to –1, then node 4 cannot be activated, because node 1 and node 2 want to activate node 4 to +1, which is contradicted by node 5, which wants to activate node 4 to –1. Then node 6 is activated to –1 persuaded by node 5. However, if node 4 is the first to be activated, then node 5 cannot be activated, and node 6 is activated to +1. Compared to the classic LT, the signed network entails more unpredictability and the process is not path-independent.

### Linear threshold model and structural balance

A series of studies have analyzed how network structure influences diffusion through the network. Centola et al. [43] studied complex propagation on random, regular and small-world networks, and pointed out that random links connected to distant nodes can prevent contagions compared to a regular lattice. This contradicted the results for cascades with simple propagation [44]. For a finite graph, the contagion has a critical threshold *T*_*c*_, and the active nodes in the stationary configuration will make up most of the system when their threshold is smaller than *T*_*c*_, while the number of active nodes will be very small when their threshold is bigger than *T*_*c*_.

For a random network with the same degree 〈*k*〉 the critical threshold will be approximately $T_{c}^{r}=1/\langle k\rangle$ [45]. According to Morris [46], the critical threshold for a regular one-dimensional lattice will be 1/2, while that for a two-dimensional lattice with near and next nearest neighbors will be 3/8. Further, Centola et al. pointed out that the critical threshold for a small-world network will lie between the values for regular and random networks [43].

**Fig 4:**
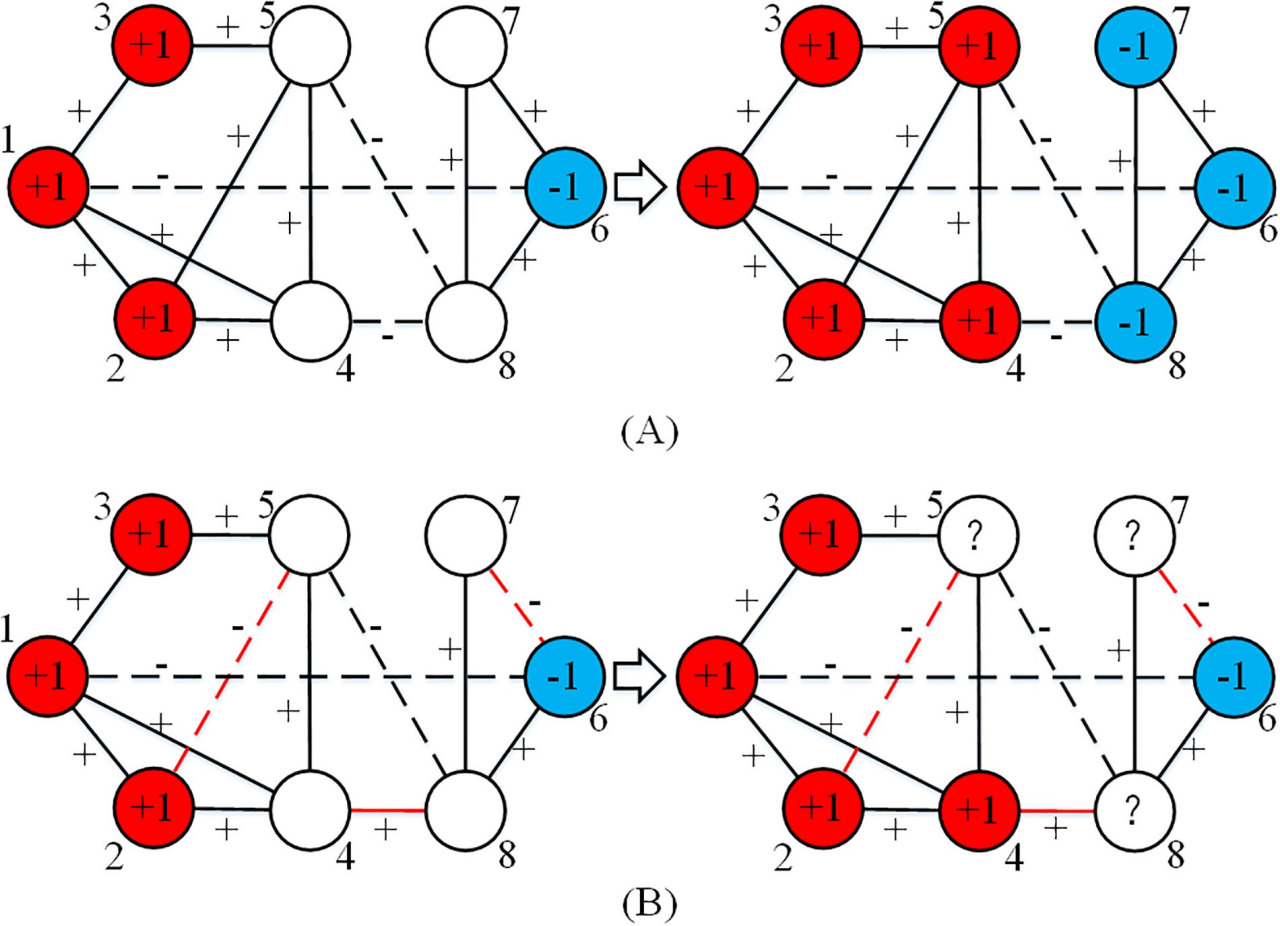
Diffusion in special structures of signed networks. Red nodes represent active +1 nodes, blue nodes represent active –1 nodes, white ones represent inactive nodes. The diffusion result in (A) is predictable, while the diffusion result in (B) is unpredictable.

In studying the effect of network structure on contagions in signed networks, it is difficult to define a specific number for the critical threshold because of the unpredictability discussed in Section 2. Moreover, it is hard to determine how to assign edge signs (+1 and –1) on a random or a regular network to form a completely random signed network or a regular signed network. This is because edge signs and edge positions are not in the same dimension. For example, given a non-negative random network, if we randomly assign signs on the network based on a uniform Bernoulli distribution multiple times, the characteristics of the resulting networks can be quite different. If more positive or negative signs are assigned to edges with greater betweenness centrality, then the generated network is definitely not a random signed network. However, some kinds of signed network structure may indeed influence the diffusion. For example, for the diffusion on the network in Fig 4(A), the inactive nodes will be activated as the figure shows, and this will be predictable. But for the diffusion on the network in Fig 4(B), in which three edges have different signs from Fig 4(A), then only node 4 is certain to be activated to +1, while the other nodes’ states cannot be predicted.

**Fig 5:**
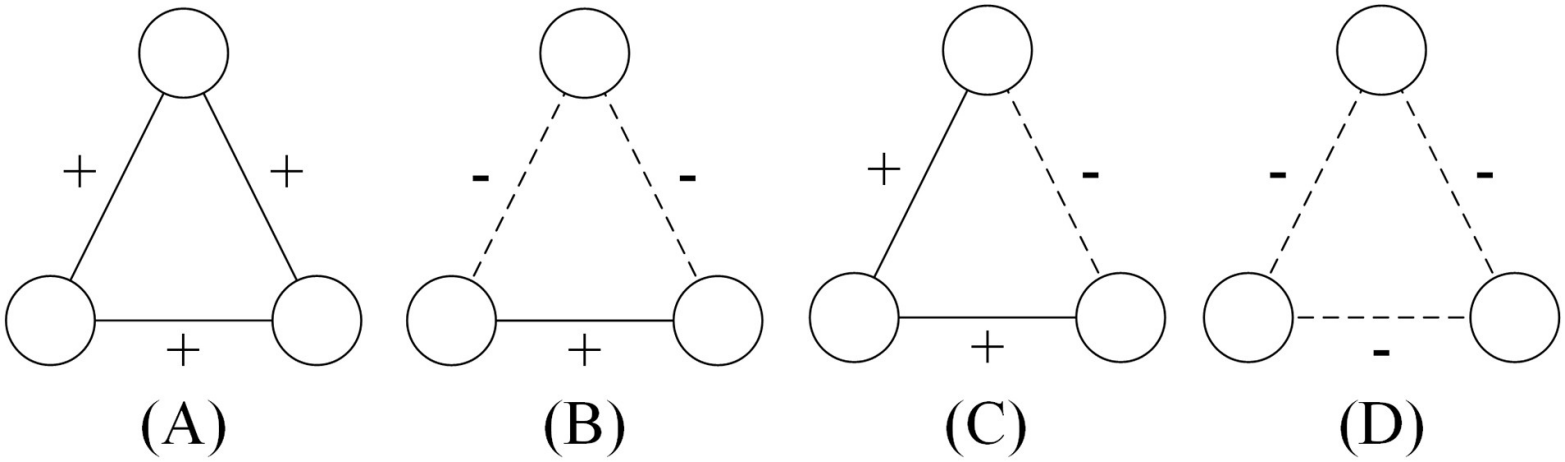
All triangles in signed networks. (A) and (B) are balanced networks, while (C) and (D) are imbalanced networks according to Heider’s definition of structural balance.

**Fig 6:**
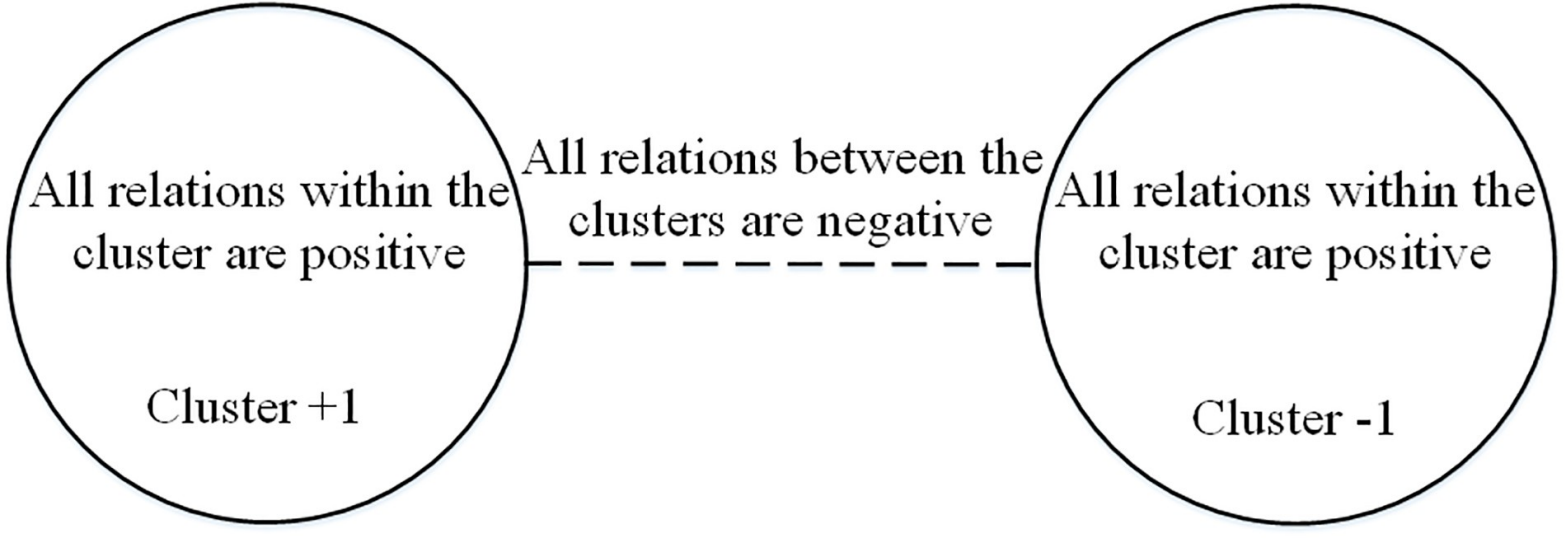
Global view of a balanced network structure according to Cartwright and Harary.

Actually, the network in Fig 4(a) is a balanced network. Heider first defined structural balance to detect the origin of conflicts in networks. According to his theory, a complete signed network is balanced if and only if every triangle has an even number of negative edges [47]. Fig 5 shows all possible kinds of triangles; those in Fig 5(A) and 5(B) are balanced, and conform to “the friend of my friend is also my friend” and “the enemy of my enemy is my friend”, while the triangles in Fig 5(C) and 5(D) are imbalanced and do not conform to this logic of friendship. Using graph theory, Cartwright and Harary generalized Heider’s theory to arbitrary networks, according to which a balanced network can be divided into two clusters: all pairs of nodes belonging to the same cluster are connected by positive edges and all pairs of nodes belonging to different clusters are connected by negative edges as shown in Fig 6 [48–50].

The LT in signed networks reflects homophily where active nodes with +1 (–1) will have a tendency to persuade their friends to take the action +1 (–1), while pushing their enemies to the cluster of –1 (+1) [35]. When the network structure is balanced and the initialized active nodes are assigned to a balanced state (nodes with the same state are connected by positive edges, while those with different states are connected by negative edges), the path of information diffusion can be predictable and this model tends to activate nodes in the direction of cluster assignation of structural balance, i.e. the active state of a node *s*_*i*_ will gradually evolve to its cluster assignation in structural balance *c*_*i*_. Then all nodes will become active on a balanced network if the threshold $T\leq \text{min}\frac{1}{\left(N_{pv}+\lambda \cdot N_{nv}\right)}$, where *N*_*pv*_ is the number of *v*’s positive-link neighbors, and *N*_*nv*_ is the number of *v*’s negative-link neighbors. The final active +1 (–1) nodes will connect positive edges with each other, while connecting negative edges with active –1 (+1) nodes.

Here we give a simple demonstration of this claim. When the threshold $T\leq \text{min}\frac{1}{\left(N_{pv}+\lambda \cdot N_{nv}\right)}$, only one edge can make an active node activate a neighboring node. If there is an inactive node in a balanced network, its positive-link neighbor will persuade it to take the same action, while its negative-link neighbor will push it to take the opposite action. As a result, all nodes can be activated if there are no isolated nodes. On the other hand, if there is a pair of active +1 (–1) nodes connected by a negative edge, they should belong to two opposing clusters according to the definition of structural balance; then there must be another node to activate one of these two nodes. If the node is a +1 (–1), this node will link positive paths to both +1 (–1) nodes. Then there will be positive links between the two clusters, which is contradicted by the structural balance. If the node is a –1 (+1) node, this node will link negative paths to both +1 (–1) nodes. Then there will be negative links within one of the clusters, which also contradicts the structural balance. If there exists a positive edge connecting a +1 node and a –1 node, they should belong to the same cluster according to the definition of structural balance, while another +1 node will link a positive path with the +1 node and link a negative path with the –1 node, which means there are negative links within the cluster; or another –1 node in another cluster will link a negative path with the +1 node and a positive path with the –1 node, which means there are positive links between the two clusters. As a result, active nodes with the same state must connect positive edges with each other, while inactive nodes with different states must connect negative edges with each other.

## Results/Discussion

Here, we explore the diffusion model in signed networks via a set of simulation experiments. These simulations are carried out by MATLAB on a 2.40 GHz CPU and 4.00 GB Memory computer, Windows 10. The experiment for each parameter set is carried out for 50,000 iterations and all experiments converge within 50,000 iterations.

We first generate an Erdős-Rényi random graph consisting of 1,000 nodes with average network connectivity 〈*k*〉 = 8 [49]. Then the signs “+” or “–” are randomly assigned to the edges according to the uniform Bernoulli distribution, making the positive-link degree 〈*k*_+_〉 = 4 and negative-link degree 〈*k*_−_〉 = 4. The simulations are carried out on this random network for all trials. We randomly select a focal node to be activated with all its positive-link neighbors being activated to +1, and all its negative-link neighbors being activated to –1. The fraction of the initial active nodes makes up less than 1%. For each parameter, the initialization operation is carried out ten times in order to cover the different situations for the initial active state, while the experiment for each initialization is carried out for 50 trials with different diffusion paths (different order of activated nodes) to explore the dynamic process.

**Fig 7:**
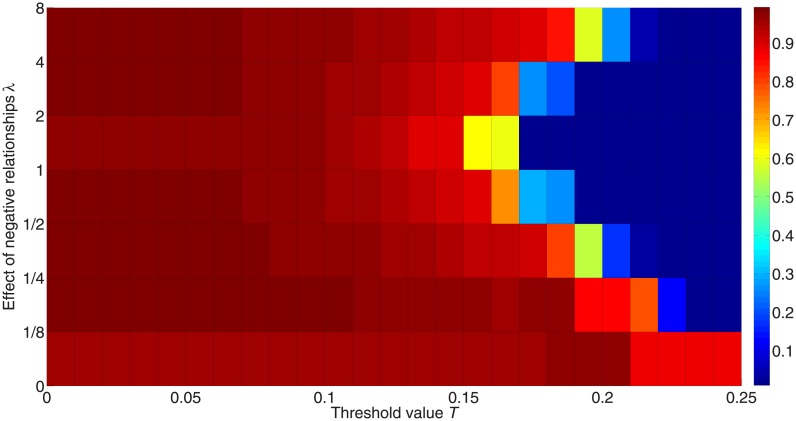
Fraction of activated nodes with different weights for the impact of negative links. 50 simulations are carried out for 50,000 iterations with 10 different initialization states on a random network consisting of 1,000 nodes with positive-link degree 〈*k*_+_〉 = 4 and negative-link degree 〈*k*_−_〉 = 4. The x-axis orders the threshold value from 0 to 0.25, while the y-axis orders the effect of negative edges on the diffusion. Colors in the figure represent the fraction of activated nodes, with red denoting a higher proportion and blue denoting a lower proportion.

Fig 7 shows the effects of negative links *λ* on the diffusion. The colors represent the fraction of activated nodes after 50,000 iterations; red represents a higher fraction, while blue represents a lower fraction. According to Watts [45], a positive-link network with degree 〈*k*〉 = 4 will have its critical threshold at 1/〈*k*〉 = 0.25. However, when more negative edges are added to the network, the cascade cannot be achieved easily with this threshold. The highest critical threshold value in this network is 0.20 if *λ* > 0 as shown in Fig 7. This shows that negative links introduce more randomness and individuals have more choices on being activated, which may give contradictory information to the neighboring nodes.

Moreover, Fig 7 shows that the threshold value undergoes an inverted U-type increase as the weight of the impact of negative links *λ* increases. When the impact of negative links equals that of positive links, i.e. *λ* = 1, the system is most unlikely to achieve information diffusion. To some extent, the diffusion based on positive links contradicts that based on negative links.

**Fig 8:**
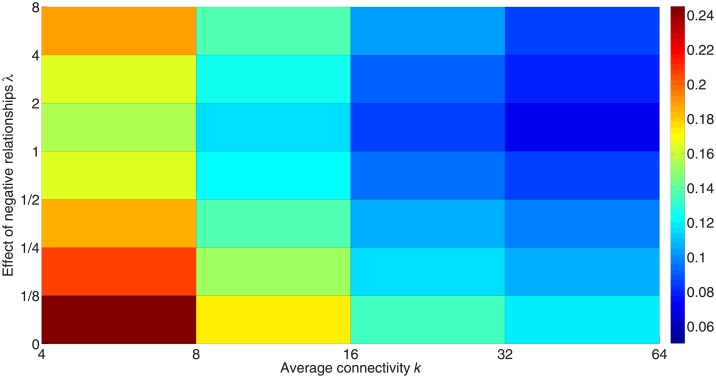
Critical thresholds with different weights for the impact of negative links and different average connectivity. Simulations are carried out on networks with different average connectivity. These networks consist of 1,000 nodes with equal positive-link and negative-link degrees. The connectivity for each network is set as <*k>* = 4, 8, 16, 32, and 64, respectively. The color in the figure represents the value of the critical threshold. Red denotes a higher value and blue denotes a lower value.

Fig 8 presents the highest critical threshold for different average connectivity of generated signed networks and different *λ*. Consistent with the result in Fig 7, the highest critical threshold with *λ* = 1 is more likely to be lower than those with other *λ*. On the other hand, a network with greater average connectivity has a lower critical threshold, which is consistent with the result in [45].

**Fig 9:**
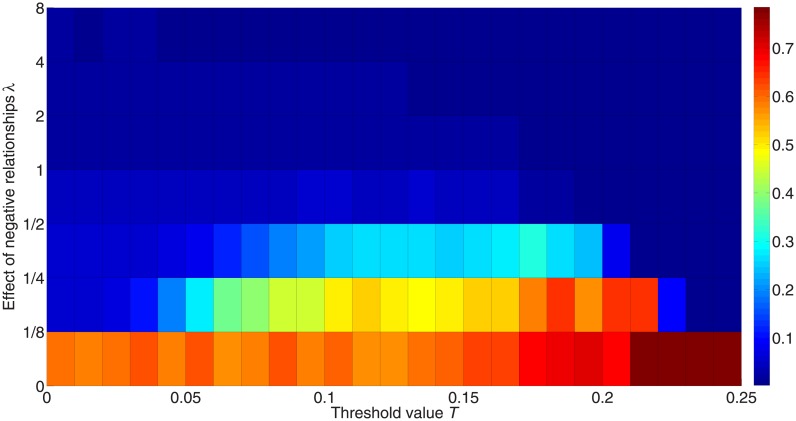
Inequality between the two active states with different weights for the impact of negative links. The color in the figure represents the difference between fractions of activated +1 and –1 nodes. Red denotes higher inequality and blue denotes lower inequality.

We here give a more specific discussion on the difference between activated +1 and –1 nodes that represents inequality between the two states, and we use the absolute value of the fraction of activated +1 nodes minus that of –1 nodes. Fig 9 shows the inequality between the two states for different *λ*, and we see that reducing the effect of negative relationships can increase the inequality between the two active states. Since positive links can activate positive-link neighbors to the same state, it is more likely to form a +1→+1→+1 or a –1→–1→–1 cascade. But a negative link can break this cascade and it is more likely to form the diffusion +1→–1→+1 or –1→+1→–1, which can make the two active states more equal.

**Fig 10:**
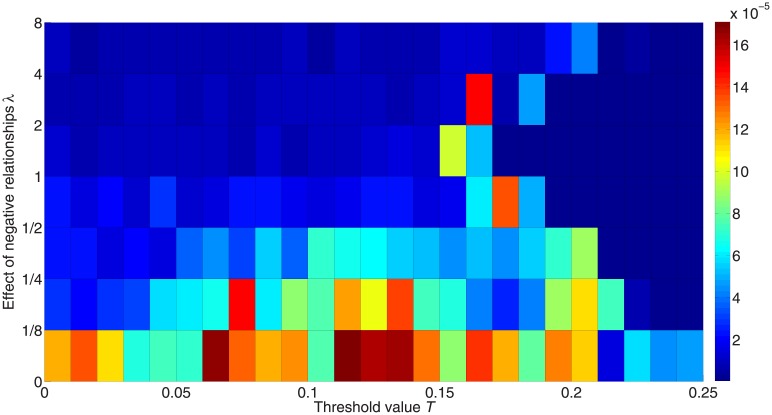
Unpredictability for different weights assigned to the impact of negative links. The color in the figure represents the unpredictability. Red denotes higher unpredictability and blue denotes higher predictability.

We here explore the predictability or unpredictability of the dynamic process. For each state *s* and initialization setting *r*, the unpredictability can be formulated as $u_{s,r}=\sum_{i=1}^{W}\sum_{j=i+1}^{W}\left|F_{s,i,r}-F_{s,j,r}\right|/\left(\begin{array}{c} W\\ 2 \end{array}\right)$ where *F*_*s*,*i*,*r*_ represents the fraction of nodes in state *s* in trial *i* with the initial condition *r* and *W* = 50 represents the number of trials [51]. The average unpredictability for each state is computed as $u_{s}=\sum_{r}^{R}u_{s,r}/R$ where *R* = 10 is the number of different initialization settings. The overall unpredictability $U=\sum_{s}^{S}u_{s}/S$ is then the average value of the unpredictability over all kinds of states including *s* = 0 (not being activated), *s* = +1 (being activated to +1), *s* = −1 (being activated to –1) and *S* = 3. Fig 10 shows the unpredictability for different *λ*, and we see that the value of each critical threshold is unpredictable. Also, a lower effect of negative relationships can decrease the predictability.

**Fig 11:**
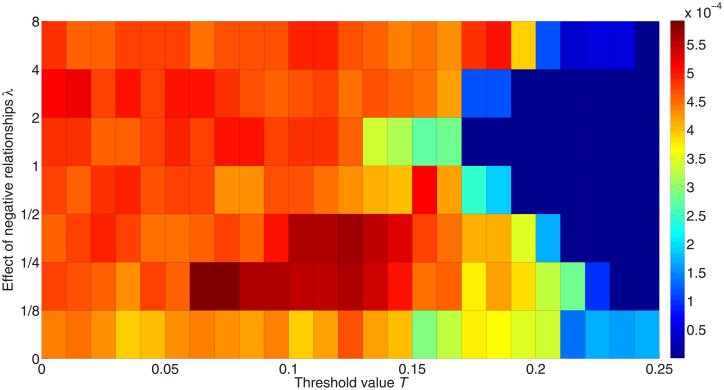
Inconsistency of results for different weights assigned to the impact of negative links. The color in the figure represents the inconsistency. Red denotes higher inconsistency and blue denotes higher consistency.

The mechanism of path dependence must be tested with many experiments with different paths, and if all of the trials with different diffusion paths result in a consistent result, then the process is path-independent. We compute here the inconsistency between the different trials in order to explore the problem of path-dependence. For each initialization setting *r*, the inconsistency between different trials can be formulated as $p_{r}=\sum_{i=1}^{W}\sum_{j=i+1}^{W}G_{r,ij}/\left(\begin{array}{c} W\\ 2 \end{array}\right)$, where *G*_*r*,*ij*_ represents the fraction of nodes with inconsistent states in trials *i* and *j* with the specific initial condition *r*, and *W* = 50 represents the number of trials. The overall inconsistency $P=\sum_{r}^{R}p_{r}/R$ is the average value over different initial conditions. When *P* equals 0, the process can be claimed to be independent. Fig 11 shows the inconsistency for different *λ*, and we see that results tend to be more consistent as the threshold increases. Compared with Fig 7, we can conclude that even though most nodes can be activated below the critical threshold, nodes may be activated to different states from different diffusion paths, which means the signed network cannot remove the path dependence no matter what the value of *λ* is.

**Fig 12:**
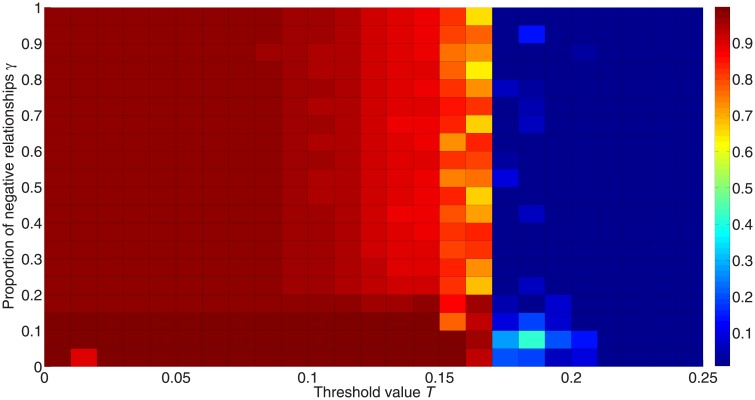
Fraction of activated nodes for different proportions of negative links. The simulation is carried out 50 times for 50,000 iterations with 10 different initialization states on random networks consisting of 1,000 nodes with degree 〈*k*〉 = 8 and *λ* = 1. The x-axis orders the threshold value from 0 to 0.25, while the y-axis orders the proportion of negative edges. The colors in the figure represent the fraction of activated nodes. Red denotes a higher proportion and blue denotes a lower proportion.

We explore the impact of the proportions of negative and positive links in signed networks on the diffusion process. Fig 12 shows the fraction of activated nodes for different proportions of negative links *γ*; we see that positive links are more likely to activate nodes, but the critical threshold value shows no differences between different values of *γ*.

**Fig 13:**
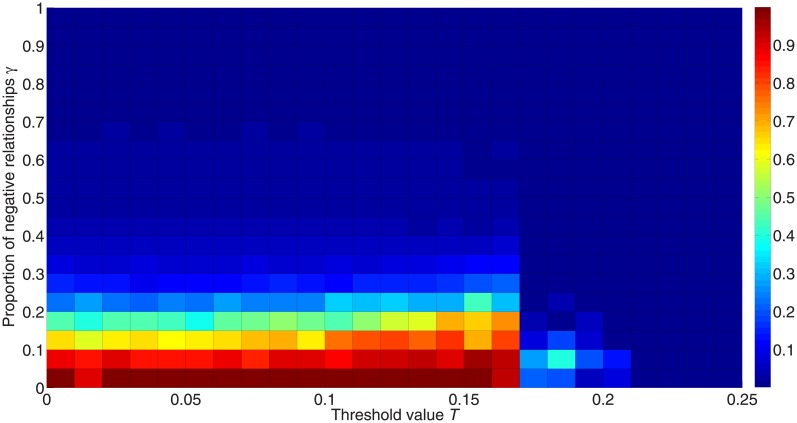
Inequality between the two active states for different proportions of negative links. The colors in the figure represent the differences between fractions of activated +1 and –1 nodes. Red denotes higher inequality and blue denotes lower inequality.

Fig 13 shows the inequality between the two active states for different values of *γ*. We see that more positive relationships can increase the inequality between the two active states, similarly to Fig 9.

**Fig 14:**
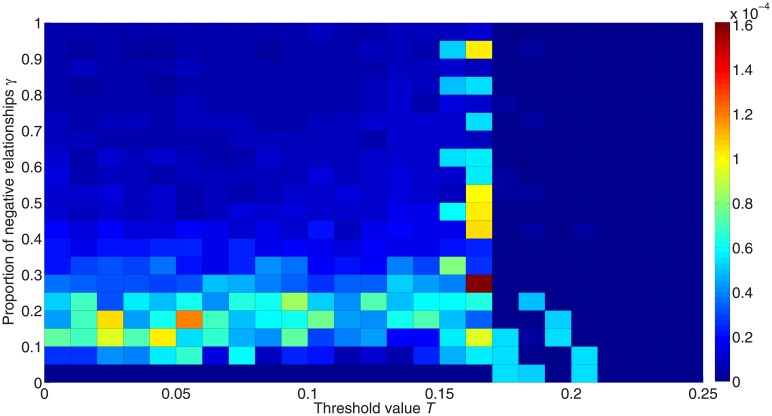
Unpredictability for different proportions of negative links. The colors in the figure represent the unpredictability. Red denotes higher unpredictability and blue denotes higher predictability.

Fig 14 presents the unpredictability for different values of *γ*. Similarly to the result in Fig 10, the critical threshold point is more unpredictable, while a greater proportion of negative links helps to improve predictability. It should be noted that for the bottom line in Fig 14 there are only positive edges in the network, so the unpredictability will always fall to 0. Since we set the maximum number of iterations at 50,000, some of the points may not actually reach 0, but may reach 0 theoretically if the maximum number of iterations were large enough.

**Fig 15:**
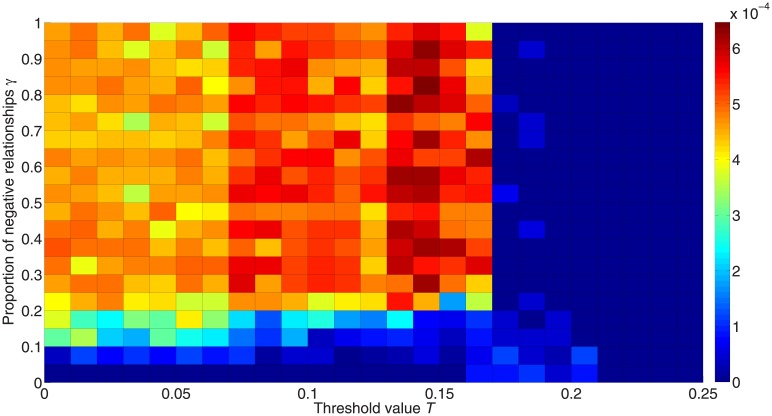
Inconsistency of results for different proportions of negative links. The color in the figure represents the inconsistency. Red denotes higher inconsistency and blue denotes higher consistency.

Fig 15 shows the inconsistency of different trials for different values of *γ*. The inconsistency increases as the threshold increases until it reaches a critical threshold. A greater proportion of negative edges decreases the consistency of different experimental trials, which means that negative edges are more likely to generate path-dependence. From this, we can conclude that it is the negative links that make the signed system path-dependent, while positive links can help remove the path-dependence as in the classic LT model.

In order to explore the influence of structural balance on the diffusion dynamics, we ran the simulations on our designed networks. The generated networks are the extension of Newman’s proposed benchmark networks [52], and the new design consists of 1,000 nodes belonging to two clusters (500 nodes per cluster) with average positive-link degree 4 and negative-link degree 4. We introduce a parameter *β* which denotes the proportion of imbalanced edges (negative links within the same cluster or positive links between different clusters). Similarly to the previous initialization, we randomly select a focal node to be activated with all its positive-link neighbors being activated to +1, and all its negative-link neighbors being activated to –1. For each parameter, the initialization operation is carried out 10 times in order to cover the different situations for the initial active state. The experiment for each initialization is carried out for 50 trials to explore the dynamic process.

**Fig 16:**
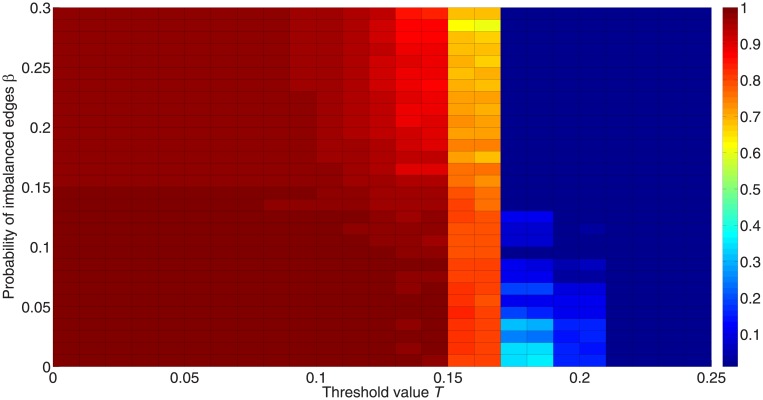
Fraction of activated nodes for different proportions of imbalanced edges. 50 simulations are carried out for 50,000 iterations with 10 different initialization states and *λ* = 1 on networks with different proportions of imbalanced edges. All these networks remain unchanged, and only some of the network signs are changed in order to generate an environment with different probabilities of imbalanced edges. These networks consist of 1,000 nodes with positive-link degree 〈*k*_+_〉 = 4 and negative-link degree 〈*k*_−_〉 = 4. The *x*-axis in this figure orders the threshold value from 0 to 0.25, while the *y*-axis orders the proportion of imbalanced edges from 0 to 0.3. The color in the figure represents the fraction of activated nodes. Red denotes a higher proportion and blue denotes a lower proportion.

Fig 16 shows the fraction of active nodes for different proportions of imbalanced edges *β*. As *β* increases, it becomes harder for networks to diffuse information, as can be seen from a lower fraction of active nodes with a higher *β*. When the network is balanced (*β* = 0), all nodes can be activated when the threshold value is smaller than 0.15, and they can be polarized from two opposing groups taking the same action. Therefore, a balanced structure allows information to spread more easily and cause polarization.

**Fig 17:**
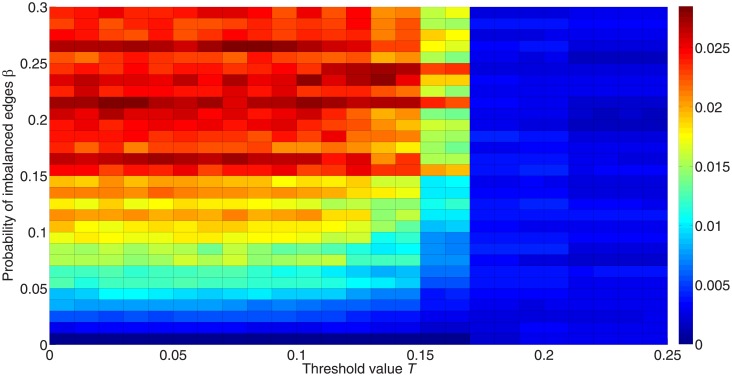
Inequality between the two active states for different proportions of imbalanced edges. The colors in the figure represent the difference between fractions of activated +1 and –1 nodes. Red denotes higher inequality and blue denotes lower inequality.

Fig 17 shows the inequality between the two active states for different *β*. We find that a lower *β* makes the two states more equal. Actually, this occurs because the network consists of two clusters with the same number of nodes, and each cluster may have only one kind of active state when the network is balanced. Thus if the two clusters have different numbers of nodes, the balanced network can still retain high inequality. What we want to show here is not that balance can increase the equality, but that balance may cause polarization, where nodes in the same clusters are activated to the same state while nodes in different clusters can be activated to different states.

**Fig 18:**
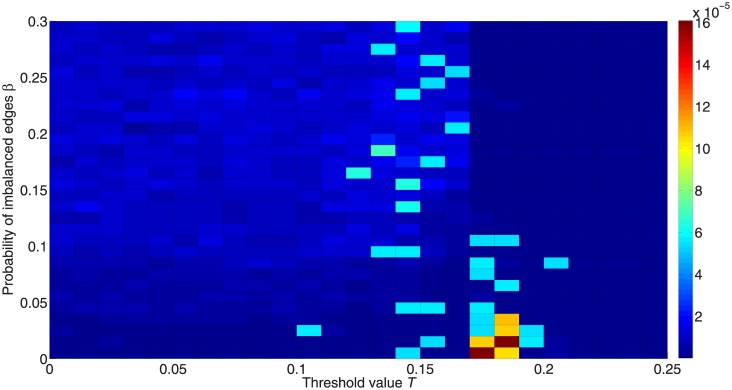
Unpredictability for different proportions of imbalanced edges. The color in the figure represents the unpredictability. Red denotes higher unpredictability and blue denotes higher predictability.

Fig 18 shows the unpredictability for different *β*. Below the critical threshold point, the dynamics with a more balanced structure can be more predictable, while above the critical threshold point, some values in more balanced networks have higher unpredictability. This is because some points in a balanced structure that can be activated have not yet been activated in some experiments within 50,000 iterations. If there were no limit on the maximum number of iterations, diffusion in a balanced structure should be exactly predictable. On the other hand, we can conclude that it is harder for a more balanced structure to become completely predictable above the critical threshold given a finite time, i.e. it would be harder for a more balanced network than for a less balanced network to mix the dynamics among nodes when the threshold is above the critical threshold. In other words, those nodes that can be activated are sometimes difficult to activate because the combined effect of structural balance and threshold surpasses the critical ones; e.g., one node can only be activated after some other specific nodes become activated, while if one of these other nodes has not been activated, the focal node cannot be activated either.

**Fig 19:**
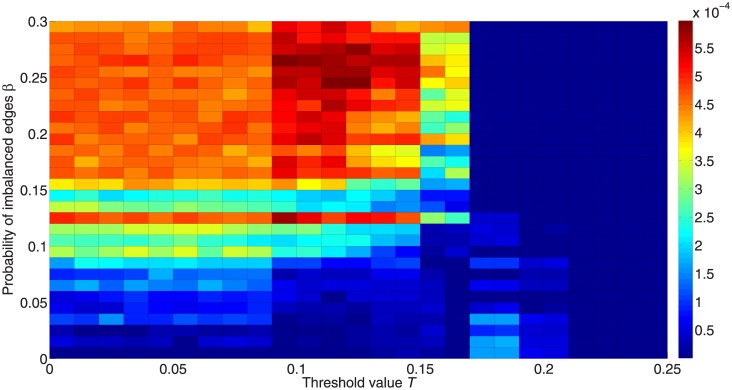
Inconsistency of results for different proportions of imbalanced edges. The color in the figure represents the inconsistency. Red denotes higher inconsistency and blue denotes higher consistency.

Fig 19 shows the inconsistency of different trials for different values of *β*. A greater proportion of imbalanced edges decreases the consistency, while a more balanced structure can help remove the path-dependence. This is consistent with the theoretical analysis in Section 3.

**Fig 20:**
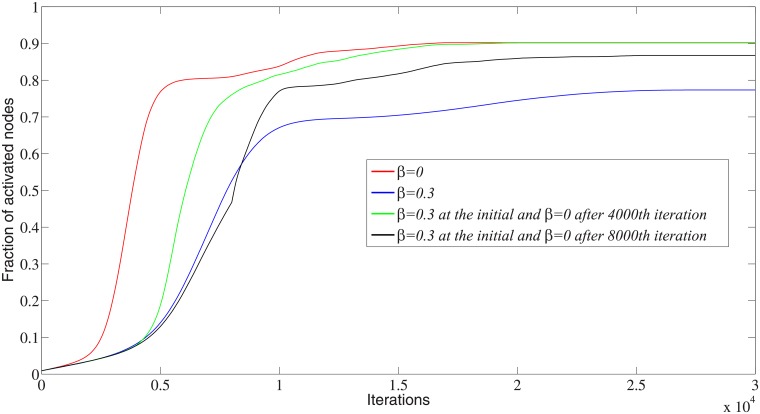
Comparison of the fraction of activated nodes for each iteration with different proportions of imbalanced edges. 50 simulations are carried out for 50,000 iterations with 10 different initialization states and *λ* = 1, *T* = 0.15. Here we present only 30,000 iterations because all curves have converged within 30,000 iterations.

We further track the evolution for each iteration with different proportions of imbalanced edges. The fraction of cooperators for each iteration in the experiment with *λ* = 1 and *T* = 0.15 is shown in Fig 20, where we see that no matter what the proportion of imbalanced edges *β*, the information diffusion will experience an S-type change in which the number of activated nodes increases slowly during the first several iterations and sharply increases at some point, after which its growth gradually slows down and eventually converges.

Comparing the curves with *β* = 0 and *β* = 0.3, the final number of activated nodes in the balanced network is larger than that in the imbalanced network. The rate of increase with *β* = 0 is also much greater than with *β* = 0.3. In this case, we conclude that the balanced network structure will increase both the magnitude and speed of the information diffusion. Moreover, we consider the changes in social ties, where the imbalanced edges are adjusted to balanced ones after the 4,000^th^ iteration (the start point of the sharp increase with *β* = 0.3) and the 8,000^th^ iteration (the end point of the sharp increase with *β* = 0.3). For the curve with *β* = 0.3 initially and *β* = 0 after the 4,000th iteration, the number of activated nodes can reach the magnitude of the curve with *β* = 0 initially, even though it may delay the diffusion of information. The experiment with *β* = 0.3 initially and *β* = 0 after the 8,000^th^ iteration activates more nodes compared to the experiment with unchanged imbalanced edges. However, it does not reach the level of *β* = 0 initially or *β* = 0 after the 4,000^th^ iteration. This is because during the first 8,000 iterations some nodes are activated to some attributes, which contradicts the diffusion in balanced networks.

## Conclusion

We have generalized the earlier linear threshold model in signed networks. We discuss the difference between the classic linear threshold model and the proposed model, and analyze the impact of network structures on the diffusion of information in signed networks. A signed network may generate more randomness, which has the opposite effect of positive links, and thus generates path dependence. Structural balance may affect information diffusion in signed networks, and a balanced network seeded with a balanced initialized active state can remove the path dependence. Simulation experiments show that when the effect of negative links is the same as that of positive links, it is more difficult to achieve information diffusion. A greater proportion of positive links in signed networks is more likely to activate nodes and remove path dependence, but positive edges reflect a +1→+1→+1 or a –1→–1→–1 diffusion mechanism, which can make the active states less equal. Moreover, based on the predictability analysis, the critical threshold point is more unpredictable, while negative links help to improve predictability. We also explore the effect of structural balance on the dynamics and find that information spreads more easily in a balanced structure, causing more activated nodes at a higher speed. Structural balance can also help remove path dependence but may cause polarization.

Diffusion in signed networks can further explain some collective action. The balanced structure in Fig 6 is typical of social conflicts [53]. When two opposing groups are more different, the antagonism between them is strengthened, which leads to more instability. According to social identity theory, the stronger the group identity, the more willing group members are to take collective action [54]. This group identity not only improves group unity, but also promotes comparison between groups, and thus generates more conflict [55]. As a result, when an accident occurs in a population with balanced structure, the information will quickly diffuse and polarization will occur [56], which corresponds to our simulation results.

In the basic diffusion model discussed here, the effects of threshold distribution and link weight distribution are not included. The initialized activation is also an ideal operation where nodes connected by positive edges are activated to the same state and those nodes connected by negative edges are activated to different states. If this condition is not met, structural balance may not remove the path-dependence. Moreover, there may exist a non-zero probability of change in the active states in the real world. The transition between +1 and –1 and transition from active to inactive states should be also considered in future work. For example, someone participating in a collective action may quit for some reason or a consumer may change their preference to another product. Including these possibilities will produce a more realistic diffusion.
